# Peripheral TNFα elevations in abstinent alcoholics are associated with hepatitis C infection

**DOI:** 10.1371/journal.pone.0191586

**Published:** 2018-02-06

**Authors:** Natalie M. Zahr

**Affiliations:** 1 Department of Psychiatry and Behavioral Sciences, Stanford University School of Medicine, Stanford, CA, United States of America; 2 Neuroscience Department, SRI International, Menlo Park, CA, United States of America; Pennsylvania State University College of Medicine, UNITED STATES

## Abstract

Substantial evidence supports the view that inflammatory processes contribute to brain alterations in HIV infection. Mechanisms recently proposed to underlie neuropathology in Alcohol Use Disorder (AUD) include elevations in peripheral cytokines that sensitize the brain to the damaging effects of alcohol. This study included 4 groups: healthy controls, individuals with AUD (abstinent from alcohol at examination), those infected with HIV, and those comorbid for HIV and AUD. The aim was to determine whether inflammatory cytokines are elevated in AUD as they are in HIV infection. Cytokines showing group differences included interferon gamma-induced protein 10 (IP-10) and tumor necrosis factor α (TNFα). Follow-up *t-tests* revealed that TNFα and IP-10 were higher in AUD than controls but only in AUD patients who were seropositive for Hepatitis C virus (HCV). Specificity of TNFα and IP-10 elevations to HCV infection status was provided by correlations between cytokine levels and HCV viral load and indices of liver integrity including albumin/globulin ratio, fibrosis scores, and AST/platelet count ratio. Because TNFα levels were mediated by HCV infection, this study provides no evidence for elevations in peripheral cytokines in "uncomplicated", abstinent alcoholics, independent of liver disease or HCV infection. Nonetheless, these results corroborate evidence for elevations in IP-10 and TNFα in HIV and for IP-10 levels in HIV+HCV co-infection.

## Introduction

Patients with chronic HIV infection typically show elevations in plasma cytokine levels [1–4]. HIV infection of the central nervous system (CNS) appears to begin with the transmigration of peripheral HIV-infected cells (e.g., monocytes or macrophages) across the blood brain barrier [5–12] and consequent infection of microglia [13–18]. Activation of macrophages and microglia and the release of chemokines, cytokines, and neurotoxins [19] promote further HIV replication [20], trafficking of macrophages into the CNS [21], glial activation [22], altered neuronal signaling and repair processes [23–26], and ultimately, compromised neuronal integrity [27–31]. Select studies in HIV patients have reported correlations between elevated plasma cytokine concentrations and cognitive deficits [32–35]. Elevations in plasma Tumor Necrosis Factor α (TNFα) [32, 36, 37] and Interferon γ-induced Protein 10 (IP-10) [33, 38–40] are frequently reported in the HIV literature and are considered biomarkers of HIV viral load (**Table 1**provides an acronym key).

**Table 1:** Acronym key.

**AIC**	Akaike Information Criterion
**AGR**	Albumin / Globulin Ratio
**AUD**	Alcohol Use Disorder
**APRI**	AST/Platelet count Ratio Index
**CSF**	Cerebrospinal Fluid
**EGF**	Epidermal Growth Factor
**EtOH**	Ethanol
**FGF**	Fibroblast Growth Factor
**Fib-4**	Fibrosis score
**Flt3**	Fms-related tyrosine kinase 3 ligand
**GCSF**	Granulocyte Colony-Stimulating Factor
**GMCSF**	Granulocyte Macrophage Colony-Stimulating Factor
**GRO**	Growth Regulated Oncogene
**HCV**	Hepatitis C Virus
**HIV**	Human Immunodeficiency Virus
**IP-10**	IFN-γ-induced protein 10
**IFN**	Interferon
**IL**	Interleukin
**MIP**	Macrophage Inflammatory Protein
**MDC**	Macrophage-Derived Chemokine
**MFI**	Mean Fluorescence Intensity
**MAPK**	Mitogen-Activated Protein Kinase
**MCP**	Monocyte Chemoattractant Protein
**NFκβ**	Nuclear Factor kappa beta
**PDGF**	Platelet-Derived Growth Factor
**RANTES**	Regulated on Activation, Normal T cell Expressed and Secreted
**SES**	Socio-economic Status
**CD40L**	soluble CD40 ligand
**SCID**	Structured Clinical Interview for DSM-IV
**TLR-4**	Toll-like Receptor 4
**TGF**	Transforming Growth Factor
**TNF**	Tumor Necrosis Factor
**VEGF**	Vascular Endothelial Growth Factor
**VACS**	Veterans Aging Cohort Study Index

Mechanisms of neuroimmune signaling in the pathogenesis of Alcohol Use Disorder (AUD) and associated brain atrophy have been proposed based primarily on animal studies [41–46]. In mice and rats, ethanol (EtOH) has been shown to activate Toll-like receptor 4 (TLR-4)[47–49], but see [50], which activates signaling molecules (e.g., members of the P38 mitogen-activated protein kinase (MAPK) family) and downstream transcription factors such as nuclear factor kappa beta (NFκβ) [51–55], to increase production of proinflammatory cytokines [56] and oxidative stress [57]. EtOH exposure in rodents has been shown to activate microglia [56, 58, 59] and upregulate proinflammatory cytokine mRNA and protein levels (e.g., monocyte chemotactic protein-1 [MCP-1]/chemokine ligand-2 [CCL2], TNFα, and interleukin (IL)-1β [IL-1β]) in several brain regions [60, 61], including frontal cortex [62, 63], cortical mantle [64, 65], hippocampus [66–69], cerebellum [49], and amygdala [70–72]. Additional support for the involvement of neuroimmune signaling in the pathogenesis AUD includes evidence for a proinflammatory environment underlying myelin disruption in EtOH-exposed mice [47]; alcohol-preferring P rats exhibiting innately elevated MCP-1 levels in the amygdala [73]; and reductions in MCP-1 in the amygdala (via silencing RNA) associated with reduced binge drinking in the P rat [74–79].

In humans, gene expression studies evaluating postmortem brain tissue from AUD relative to healthy controls showed a strong representation of immune- and inflammation- related genes in the AUD brain [80, 81]. A number of studies have evaluated whether polymorphisms in innate immune genes (e.g., NFκβ, TNFβ) contribute to the genetic risk for alcoholism, with equivocal results [82–85] but see [86–90]. These findings were elaborated by an influential paper showing in AUD relative to control human brain tissue higher MCP-1 protein levels in the ventral tegmental area (VTA), substantia nigra, hippocampus, and amygdala, and altered microglial morphology in the cingulate cortex, VTA, and midbrain [48, 91, 92]]. In vivo, withdrawal from alcohol has been associated with higher cerebrospinal fluid (CSF) levels of MCP-1 in alcoholics relative to healthy controls [93]. Stimulation of macrophages and mononuclear cells isolated from human subjects with AUD results in augmented proinflammatory cytokine production compared to cells from healthy controls [94, 95]. Peripheral (plasma/serum) cytokines reported as elevated in AUD include IL-1β [96], IL6 [97, 98], IP-10, and MCP-1 [99–106]]. Higher than control levels of TNFα have frequently been reported [107, 108] but see [109] and associated with AUD severity [97, 110, 111] and alcohol craving at early abstinence [98].

The considerable comorbidity of HIV infection and alcoholism [112–118] negatively impacts multiple biological systems, but particularly affects the progression of liver disease [119–122], which has emerged as a major cause of morbidity and mortality among HIV-infected patients [123]. In rodent models, EtOH exposure to HIV-infected animals resulted in greater elevations in MIP-2 [124] or MCP-1 [125] than HIV infection alone. In macaque models, muscle TNFα mRNA expression was markedly increased above baseline levels at 10 months post-infection in simian immunodeficiency (SIV) + EtOH-exposed animals [126]; IFNα levels were higher in the spleen of EtOH-exposed relative to vehicle exposed SIV-infected monkeys [127]. In humans, peripheral IL-6 levels were high in HIV-infected patients with alcohol problems [128, 129].

To evaluate whether peripheral cytokines are elevated in AUD relative to the HIV phenotype, this study compared 4 groups of human participants: those with AUD or HIV, those with HIV+AUD, and those without either condition (i.e., healthy controls). Based on the extant literature, we hypothesized that **1)** HIV infection would be associated with elevated levels of IP-10 and TNFα; **2)** an AUD diagnosis would be associated with elevated levels of TNFα; and **3)** comorbidity for HIV+AUD would be associated with synergistic effects on elevating TNFα levels. Secondary analyses considered contributions to observed differences from disease-related factors, such as hematological indices of liver function.

## Methods

### Participants

This study was conducted in accordance with protocols approved by the Institutional Review Boards of Stanford University and SRI International. Written informed consent was obtained from all participants in accordance with the Declaration of Helsinki by the signing of consent documents in the presence of staff after staff ensured that each participant understood the information provided and appreciated the reasonably foreseeable consequences of a participating in the study. Study participants were healthy controls (26 women/28 men, 50.7±10.9 years), individuals with AUD (27 women/54 men, 51.1±8.8 years; currently sober as demonstrated by a negative Breathalyzer test given immediately following consent), those infected with HIV (16 women/28 men, 55.8±7.3 years), and those comorbid for HIV and AUD (16 women/28 men, 55.4±6.3 years).

AUD participants were recruited from local substance abuse treatment programs. HIV patients were referred from local outpatient or treatment centers, or recruited during presentations by project staff and by distribution of flyers at community events. Comparison participants were recruited from the local community by referrals and flyers. All participants were then screened using the Structured Clinical Interview for DSM-IV (SCID) [130], structured health questionnaires, and a semi-structured timeline follow-back interview to quantify lifetime alcohol consumption [131, 132]. Upon initial assessment, subjects were excluded if they had a significant history of medical (e.g., epilepsy, stroke, multiple sclerosis, uncontrolled diabetes, or loss of consciousness > 30 minutes), psychiatric (i.e., schizophrenia or bipolar I disorder), or neurological disorders (e.g., neurodegenerative disease) other than alcohol abuse or dependence in the AUD group. Other exclusionary criteria were recent (i.e., past 3 months) substance dependence other than alcohol in the AUD group or any DSM-IV Axis I disorder in the control group. Severity of depressive symptoms was assessed with the Beck Depression Inventory-II [133] in all groups.

**Table 2**presents demographic data for each of the 4 groups. The control and AUD groups were younger than the HIV and HIV+AUD groups (p = .0019). The 3 patient groups relative to the control group were less educated, had poorer socio-economic status (SES) [134] and global functioning (i.e., GAF) [135], scored lower on the Wechsler Test of Adult Reading (WTAR) [136] and the Dementia Rating Scale (DRS) [137], and had more depressive symptoms (as determined by the BDI-II) (all p≤.0001). The Veterans Aging Cohort Study (VACS) index, which predicts all-cause mortality, cause-specific mortality, and other outcomes in those living with HIV infection [138] was higher in the 2 HIV groups (HIV and HIV+AUD) than the control and AUD groups; the Karnofsky score, a standard to measure patients ability to perform ordinary tasks [139] was low in the HIV+AUD group relative to the 3 comparison groups.

**Table 2:** Demographic characteristics of the 4 study groups: Mean ± SD / frequency count.

	Control (n = 54)	AUD (n = 81)	HIV (n = 44)	HIV + AUD (n = 44)	p-value*
**N (men/women)**	28/26	54/27	28/16	28/16	0.4684
**Age (years)**	50.7±10.9	51.1±8.8	55.8±7.3	55.4±6.3	**0.0019**
**Education (years)**	16.1±2.4	12.9±2.4	13.8±2.3	13.1±2.1	**< .0001**
**Handedness (Right/Left/Ambidexterous)**	48/3/3	70/9/2	40/3/1	39/5/0	0.4885
**Body Mass Index**	26.9±5.0	28.2±5.0	26.2±4.8	27.1±4.5	0.2075
**Socioeconomic Status**^**a**^	25.8±11.7	45.5±15.0	38.6±14.7	44.0±12.9	**< .0001**
**WTAR IQ**	110.6±14.6	94.0±18.6	94.9±17.8	87.0±17.5	**< .0001**
**Dementia Rating Scale**	139.5±3.2	134.8±5.6	137.4±4.3	134.5±4.4	**< .0001**
**Global Assessment of Functioning**	84.9±7.0	70.6±11.2	73.9±10.6	68.7±10.5	**< .0001**
**AUD onset age**	-	24.9±9.1	-	23.9±10.4	0.5640
**Lifetime Alcohol Consumption**	32.6±40.1	1424.2±1079.8	72.3±73.9	1147.6±1023.5	**< .0001**
**Days since last Drink**	44.1±117.1	96.1±96.3	78.6±141.2	75.3±161.3	0.2229
**AUDIT scores**^**b**^	2.4±2.5	16.4±11.2	2.2±2.5	9.9±10.3	**< .0001**
**History of ER Detoxifications**^**c**^	-	13/68	-	4/40	0.225
**Withdrawal Scores**^**d**^	-	3.4±2.6	-	1.9±2.4	**0.0015**
**Beck Depression Inventory-II**	1.5±2.1	9.5±8.6	8.7±7.3	10.9±8.5	**< .0001**
**Karnofsky score**	100.0±0	99.7±2.4	99.8±1.5	98.5±4.2	**0.0366**
**VACS Index**	14.56±10.84	17.67±12.53	33.44±17.49	29.24±14.40	**< .0001**
**HIV onset age (years)**	-	-	35.9±10.0	33.6±7.3	0.2256
**HIV duration (days)**	-	-	7336.3±2785.1	8034.8±2421.0	0.2211
**CD4 cell count (100/mm**^**3**^**)**	-	-	669.9±265.3	675.7±335.4	0.2235
**CD4 cell count nadir (100/mm**^**3**^**)**	-	-	240.2±194.8	199.9±184.1	0.3867
**Viral Load (log copies/mL)**	-	-	1.7±0.9	1.9±1.1	0.3254
**AIDS-defining event (yes/no)**^**e**^	-	-	16/28	26/18	**< .0001**
**HAART (yes/no)**	-	-	40/4	40/4	0.9449
**Efavirinz, including Atripla (yes/no)**	-	-	9/35	10/34	0.7956
**Hepatitis C Virus (positive/negative)**	-	16/65	13/31	21/23	**< .0001**
**Treatment for HCV infection**^**f**^	-	4/16	4/13	5/21	0.8984
**Smoker (never/past/current)**	51/1/2	16/23/42	25/7/12	13/10/21	**< .0001**
**Self-Defined Ethnicity (Caucasian/AA)**^**g**^	44/10	40/41	26/18	14/30	**< .0001**

*4-group comparisons: ANOVA used on continuous variables (e.g., age); χ2 used on nominal variables (e.g., handedness)

^a^lower score = higher status

^b^AUDIT = Alcohol Use Disorders Identification Test

^c^Self report of visit to emergency room for alcohol-related problems.

^d^Sum of 8 possible withdrawal signs (autonomic signs, tremor, insomnia, nausea, agitation, anxiety, seizures, hallucinations)

^e^including AIDS-defining illness or CD4 prior nadir <200cells/μl

^f^Self report of HCV treatment

^g^AA = African American

### Sample collection and processing

Whole blood samples (n = 294), collected in lavender EDTA tubes between March 2013 and October 2016, were centrifuged (500 rcf at room temperature for 10min). Plasma was transferred to 1.5mL conical tubes, centrifuged at 13,000 rcf at room temperature for another 10min, and the resulting supernatant was transferred to 1.5mL conical tubes for storage at −80° C until analysis by the Human Immune Monitoring Center. Additional blood samples were collected and analyzed by Quest Diagnostics for complete blood count with differential, comprehensive metabolic panel, HIV and hepatitis C (HCV) screening, and RNA quantification when relevant (i.e., for HIV or HCV seropositive results). Quest laboratory results were missing for 11 control, 3 AUD, 1 HIV, and 3 HIV+AUD participants.

### Immunological assays

The Human Immune Monitoring Center (http://iti.stanford.edu/himc/), which continually benchmarks processes to minimize technical variability (Maecker et al., 2005), performed immunological assays. Human 41-plex kits (HCYTOMAG-60K, 7 kits, each able to run 42 samples) were purchased from EMD Millipore and used according to the manufacturer’s recommendations with modifications as described. Briefly, samples were mixed with antibody-linked magnetic beads on a 96-well plate and incubated overnight incubation at 4°C with shaking. Cold and room temperature incubation steps were performed on an orbital shaker at 500–600 rpm. Plates were washed twice with wash buffer in a Biotek ELx405 washer. Following one hour incubation at room temperature with biotinylated detection antibody, streptavidin fluorochrome (i.e., streptavidin-PE) was added for 30 minutes with shaking. Plates were washed as above and PBS added to wells for reading in the Luminex 200 Instrument with a lower bound of 50–100 beads per sample per cytokine. Each sample was measured in duplicate. Custom assay control beads by Radix Biosolutions were added to all wells.

The 41 cytokines included in each kit belong to 4 families: ***hematopoietin*** (interleukin (IL)-1α, IL-1β, IL-1RA, IL2, IL3, IL4, IL5, IL6, IL7, IL9, IL10, IL12-p40, IL12-p70, IL13, IL15, IL17, soluble CD40 ligand (CD40L), Fms-related tyrosine kinase 3 ligand (Flt3 ligand), granulocyte colony-stimulating factor (GCSF), granulocyte macrophage CSF (GMCSF)), ***chemokines*** (epidermal growth factor (EGF), eotaxin (CCL11), fibroblast growth factor (FGF)-2, fractalkine, RANTES (regulated on activation, normal T cell expressed and secreted/CCL5), growth regulated oncogene (GRO/CXCl1), IL8, Interferon-γ-induced protein 10 (IP-10/CXCL10), monocyte chemoattractant protein 1 (MCP-1/CCL2), MCP-3 (CCL7), macrophage-derived chemokine (MDC/CCL22), macrophage inflammatory protein (MIP)-1α, MIP-1β, transforming growth factor (TGF)-α, vascular endothelial growth factor (VEGF)), ***growth factors*** (platelet-derived growth factor (PDGF)-AA, PDGF-BB, Tumor Necrosis Factor α (TNF-α), TNF-β), and ***interferons*** (IFN-α2, IFN-γ).

### Liver status Assessments

We used standard laboratory results from Quest blood assays to calculate 2 noninvasive indices of liver fibrosis. The Fibrosis index (FIB-4: based on age, aspartate aminotransferase (AST), alanine aminotransferase (ALT), and platelet count) [140] and the AST/platelet count ratio (APRI) score both have high predictive accuracy for diagnoses of HCV e.g., [141, 142].

Fib-4=age(years)×AST(UL)platletcount(109L)×ALT(UL)

APRIscore=AST(IUL)/ASTupperlimitofnormal=40(IUL)plateletcount(109L)×100

### Statistical analysis

Of 294 samples, 6 individuals (1 AUD man, 2 HIV men, 1 HIV women, 1 HIV+AUD man, 1 HIV+AUD women) were excluded (e.g., low IQ, abnormal brain scan, diseases such as epilepsy or Progressive Multifocal Leukoencephalopathy). Longitudinal follow-up samples from individual subjects were also removed, yielding a total of 223 unique, single-visit samples (control n = 54, AUD n = 81, HIV n = 44, HIV+AUD n = 44). Based on a previous publication evaluating cytokine levels in AUD patients [98], and G*Power 3.1, we calculated an effect size of 3.8. Using this effect size with an alpha error probability of 0.5 and our control (n = 54) + AUD (n = 81) sample sizes, the current study was found to have a power of 1.

Based on the recommendation of the HIMC, the average of 2 readings for mean fluorescence intensity (MFI) for each analyte was used because these values have less variance than pg/mL measures (presented in **S1 Table**). In addition, corrected (studentized-residual) MFI values, based on results of an Akaike information criterion (AIC) model including kit number (nominal: 1–7), age (continuous), sex (nominal: M/F), socio-economic status (SES, continuous), and ethnicity (nominal: White/Black) were considered (**S2 Table**).

Diagnoses effects were evaluated using analysis of variance (ANOVA). Two-group comparisons used *t-tests*. Correlations were evaluated using Spearman’s ρ. Multiple regressions were used when relevant.

## Results

### 4-group differences in cytokine levels

**Fig 1:**
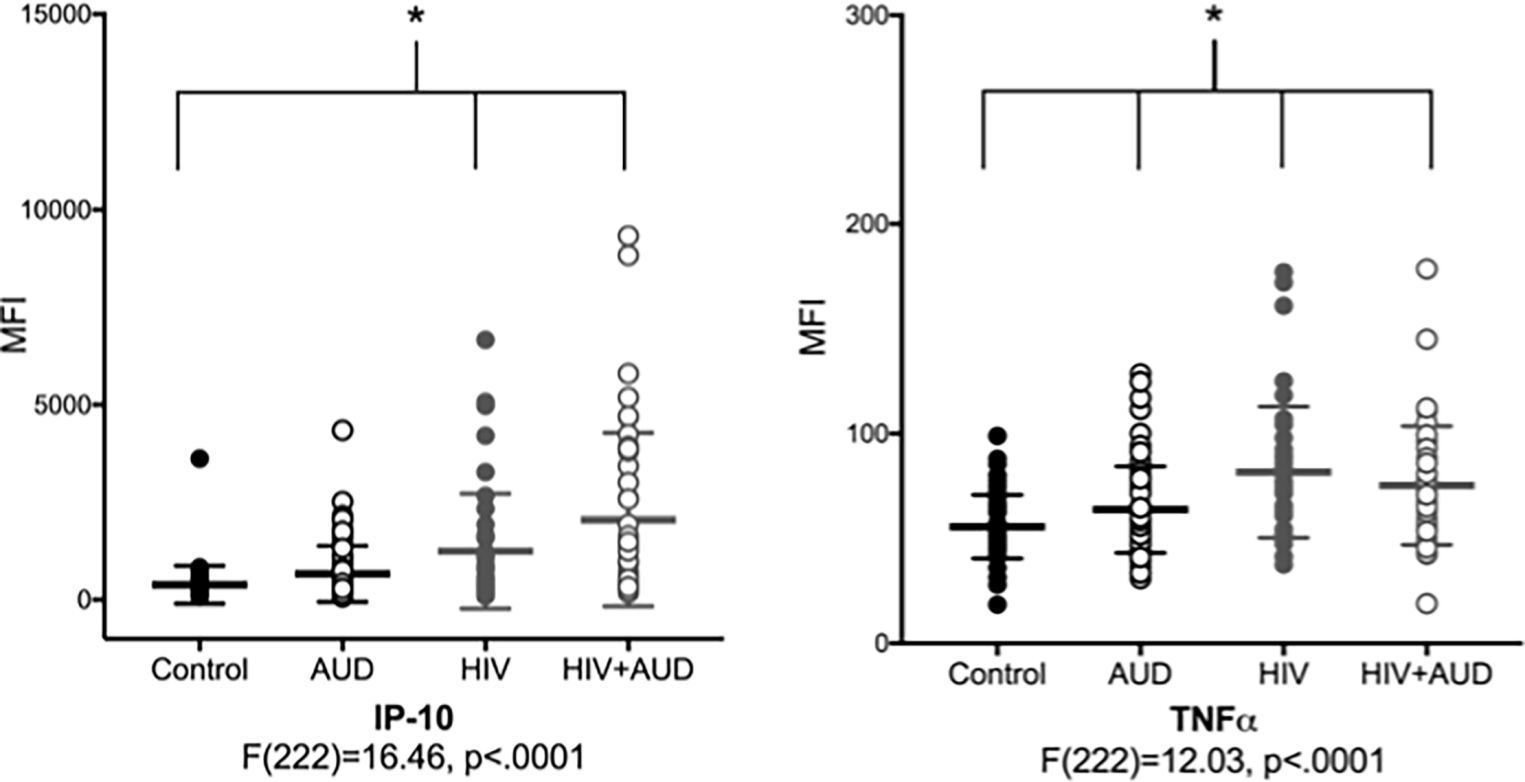
Scatter plots of the **a)** the chemokine Interferon γ-induced Protein 10 (IP-10) and the **b)** cytokine Tumor Necrosis Factor α (TNFα) in the 4 groups (Control: black closed circles; AUD: black open circles; HIV: gray closed circles; HIV+AUD: gray open circles). * indicates significance at p = .001.

Results of separate 4-group ANOVAs for each of the 41 analytes are presented in **Table 3**. Post-hoc tests indicated that the most common results were lower levels of cytokines (i.e., IL-1α, IL-1β, IL2, IL3, IL9, IL12P40, and IL13) in the HIV and HIV+AUD groups relative to the control group. Cytokines that were higher in the 2 HIV groups (i.e., HIV and HIV+AUD) relative to the control group included IP-10 and MCP-1. TNFα was high in the 3 patient groups relative to the control group (**Fig 1**). IP-10 and TNFα results were similar when studentized-residual values were considered (**S2 Table**).

**Table 3:** Cytokine levels* in the 4 study groups: Mean ± SD and ANOVA results.

cytokine	Control (n = 54)	AUD (n = 81)	HIV (n = 44)	HIV + AUD (n = 44)	ANOVA	
					F Ratio	p value
**CD40L**	68.85±51.01	139.95±267.02	204.74±923.29	62.30±44.57	1.09	0.35
**EGF**	28.56±30.31	25.64±23.01	76.67±320.78	17.09±8.44	1.61	0.19
**EOTAXIN**	81.38±55.35	92.74±79.23	127.24±101.47	103.28±81.39	2.93	**0.03**
**FGFB**	23.87±24.36	21.61±11.25	19.11±15.45	18.18±19.99	1.04	0.38
**FLT3L**	32.34±47.42	29.66±16.39	47.22±105.38	26.79±13.04	1.34	0.26
**Fractaline**	17.73±10.84	21.06±16.90	18.37±13.37	15.44±5.68	1.87	0.14
**GCSF**	32.36±21.34	26.46±10.43	24.62±10.85	28.58±22.05	2.15	0.09
**GMCSF**	26.44±10.65	37.19±100.92	22.31±5.71	23.81±9.84	0.79	0.50
**GRO**	336.12±464.17	664.88±873.39	423.09±708.46	415.28±530.30	2.89	**0.04**
**IFNA2**	20.17±12.31	26.06±36.94	18.66±8.02	18.69±6.45	1.48	0.22
**IFNG**	79.88±75.19	42.45±37.29	49.30±78.31	45.07±51.70	4.75	**0.003**
**IL10**	36.72±38.59	30.58±17.56	28.84±25.20	25.60±7.89	1.78	0.15
**IL12P40**	30.46±26.63	27.12±15.56	21.90±7.35	20.24±6.36	3.98	**0.009**
**IL12P70**	22.63±19.55	21.65±14.96	16.90±7.65	17.98±18.19	1.58	0.20
**IL13**	29.54±47.57	21.34±25.20	15.82±9.78	14.42±5.22	2.92	**0.04**
**IL15**	32.78±24.27	32.06±16.33	27.15±7.48	27.48±13.31	1.60	0.19
**IL17**	61.01±88.69	39.74±39.58	42.72±69.16	30.39±27.26	2.37	0.07
**IL1A**	32.25±25.17	28.14±13.64	24.22±6.97	23.10±5.71	3.62	**0.01**
**IL1B**	24.13±22.38	22.29±13.74	16.20±5.52	15.22±4.50	4.96	**0.002**
**IL1RA**	27.25±18.66	27.10±26.55	31.43±48.26	25.05±15.42	0.38	0.77
**IL2**	25.08±23.45	21.77±14.57	17.02±7.05	16.68±7.58	3.51	**0.02**
**IL3**	24.25±15.13	22.02±11.52	19.05±4.46	19.43±3.79	2.66	**0.05**
**IL4**	37.12±33.04	29.38±15.09	26.43±12.61	26.24±14.71	3.13	**0.03**
**IL5**	20.78±34.52	16.04±12.18	18.74±35.47	13.25±6.94	0.89	0.45
**IL6**	36.29±36.56	26.90±18.42	30.22±40.19	22.64±21.15	1.98	0.12
**IL7**	23.69±14.89	23.03±11.32	20.03±7.59	20.86±11.64	1.11	0.34
**IL8**	150.14±146.85	124.39±100.51	146.60±141.51	147.70±137.15	0.60	0.62
**IL9**	28.88±26.92	26.03±21.88	18.52±5.90	17.75±5.32	4.25	**0.006**
**IP10**	386.13±486.47	665.69±719.76	1250.73±1478.36	2057.29±2224.48	16.46	**< .0001**
**MCP1**	774.71±545.61	909.48±468.12	1112.89±876.39	1153.47±747.67	1.00	**0.01**
**MCP3**	37.76±65.63	25.46±45.38	17.65±13.32	19.57±23.56	2.12	0.10
**MDC**	776.05±414.41	861.33±452.91	835.62±467.82	900.73±471.60	0.69	0.56
**MIP1A**	61.01±47.40	53.42±112.77	47.52±29.03	259.68±1243.43	1.61	0.19
**MIP1B**	52.44±62.30	41.26±16.26	41.08±33.53	90.75±346.86	1.07	0.36
**PDGFAA**	2727.60±1991.09	3826.70±3065.67	3029.29±3467.43	2861.59±1902.72	2.24	0.08
**PDGFBB**	414.39±533.71	636.60±637.51	594.13±1598.30	447.69±414.45	0.93	0.42
**RANTES**	8942.89±4267.98	8775.41±4176.13	9362.59±3455.16	9652.91±3444.59	0.57	0.64
**TGFA**	22.27±19.39	26.54±23.24	41.36±137.76	25.21±42.41	0.77	0.51
**TNFA**	55.66±15.13	63.78±20.58	81.68±31.27	75.26±28.28	12.03	**< .0001**
**TNFB**	32.90±46.55	26.25±33.23	29.02±76.68	17.93±11.88	0.90	0.44
**VEGF**	29.74±32.42	26.19±16.79	32.13±78.11	22.09±28.51	0.51	0.67

*average of 2 mean fluorescence intensity (MFI) values per analyte

### 2-group differences in cytokine levels

For direct evaluation of single diagnoses effects on peripheral cytokine levels, additional statistics used *t-tests* to compare control and individual patient groups (**Table 4**). Table 4 also includes remaining comparisons (e.g., AUD vs. HIV; AUD vs. HIV+AUD; HIV vs. HIV+AUD). An AUD diagnosis, relative to healthy controls, was associated with higher levels of CD40L, GRO, PDGFAA, PDFGBB, IP-10, and TNFα and lower levels of IFN-γ and MIP-1α. This pattern of cytokines associated with an AUD diagnosis was significantly different from that presenting in HIV infection. In HIV relative to healthy controls, EGF, MCP-1, IP-10, and TNFα levels were high and GCSF, GMCSF, IL-1α, IL-1β, IL2, IL3, IL4, IL9, IL12p40, IL13, MCP-3, and TNFβ were low. The results relative to controls in the comorbid HIV+AUD group were very similar to those in the HIV only group: MCP-1, IP-10, and TNFα levels were high and EGF, FGFB, IFN-y, IL-1α, IL-1β, IL2, IL3, IL4, IL6, IL9, IL10, IL12p40, IL13, IL17, and TNFβ were low. In comparing HIV relative to HIV+AUD, only IP-10 was significantly different between groups, and was higher in HIV+AUD relative to HIV only. The only cytokines that were affected in all 3 (individual) patient groups relative to controls were IP-10 and TNFα. Results of 2-group comparisons were circumscribed when studentized-residual values were evaluated: relative to healthy controls, only TNFα levels were high in AUD and only IP-10 and TNFα levels were high in HIV or HIV+AUD (**S3 Table**).

**Table 4:** Two-group t-test* comparisons of cytokine levels.

cytokine	*Con*. *vs*. *AUD*		*Con*. *vs*. *HIV*		*Con*. *vs*. *HIV+AUD*		*AUD vs*. *HIV*		*AUD vs*. *HIV+AUD*		*HIV vs*. *HIV+AUD*	
	t Ratio	p value	t Ratio	p value	t Ratio	p value	t Ratio	p value	t Ratio	p value	t Ratio	p value
**CD40L**	2.33	**0.02**	n.s.		n.s.		n.s.		-2.55	**0.01**	n.s.	
**EGF**	n.s.		2.69	**0.009**	-2.66	**0.01**	n.s.		-2.99	**0.003**	n.s.	
**EOTAXIN**	n.s.		n.s.		n.s.		n.s.		n.s.		n.s.	
**FGFB**	n.s.		n.s.		-2.25	**0.03**	n.s.		n.s.		n.s.	
**FLT3L**	n.s.		n.s.		n.s		n.s.		n.s.		n.s.	
**Fractaline**	n.s.		n.s.		n.s.		n.s.		n.s.		n.s.	
**GCSF**	n.s.		-2.32	**0.02**	n.s.		n.s.		n.s.		n.s.	
**GMCSF**	n.s.		-2.45	**0.02**	n.s.		n.s.		n.s.		n.s.	
**GRO**	2.84	**0.005**	n.s.		n.s.		n.s.		-1.99	**0.05**	n.s.	
**IFNA2**	n.s.		n.s.		n.s.		n.s.		n.s.		n.s.	
**IFNG**	-3.39	**0.001**	n.s.		-2.71	**0.008**	n.s.		n.s.		n.s.	
**IL1A**	n.s.		-2.24	**0.03**	-2.59	**0.01**	-2.13	**0.04**	-2.89	**0.005**	n.s.	
**IL1B**	n.s.		-2.51	**0.01**	-2.86	**0.006**	-3.50	**0.0007**	-4.23	**< .0001**	n.s.	
**IL1RA**	n.s.		n.s.		n.s.		n.s.		n.s.		n.s.	
**IL2**	n.s.		-2.40	**0.02**	-2.48	**0.02**	-2.45	**0.02**	-2.57	**0.01**	n.s.	
**IL3**	n.s.		-2.40	**0.02**	-2.25	**0.03**	-2.06	**0.04**	n.s.		n.s.	
**IL4**	n.s.		-2.19	**0.03**	-2.17	**0.03**	n.s.		n.s.		n.s.	
**IL5**	n.s.		n.s.		n.s.		n.s.		n.s.		n.s.	
**IL6**	n.s.		n.s.		-2.31	**0.02**	n.s.		n.s.		n.s.	
**IL7**	n.s.		n.s.		n.s.		n.s.		n.s.		n.s.	
**IL8**	n.s.		n.s.		n.s.		n.s.		n.s.		n.s.	
**IL9**	n.s.		-2.75	**0.008**	-2.97	**0.004**	-2.90	**0.005**	-3.23	**0.002**	n.s.	
**IL10**	n.s.		n.s.		-2.07	**0.04**	n.s		-2.18	**0.03**	n.s.	
**IL12P40**	n.s.		-2.26	**0.03**	-2.73	**0.008**	-2.54	**0.01**	-3.48	**0.0007**	n.s.	
**IL12P70**	n.s.		n.s.		n.s		-2.35	**0.02**	n.s.		n.s.	
**IL13**	n.s.		-2.07	**0.04**	-2.32	**0.02**	n.s		-2.38	**0.02**	n.s.	
**IL15**	n.s.		n.s.		n.s		-2.23	**0.02**	n.s.		n.s.	
**IL17**	n.s.		n.s.		-2.40	**0.02**	n.s		n.s.		n.s.	
**IP10**	2.69	**0.008**	3.72	**0.0005**	4.89	**< .0001**	2.47	**0.02**	4.04	**0.0002**	2.00	**0.05**
**MCP1**	n.s.		2.23	**0.03**	2.81	**0.006**	n.s.		n.s.		n.s.	
**MCP3**	n.s.		-2.20	**0.03**	n.s.		n.s.		n.s.		n.s.	
**MDC**	n.s.		n.s.		n.s.		n.s.		n.s.		n.s.	
**MIP1A**	-2.90	**0.005**	n.s.		n.s.		n.s.		n.s.		n.s.	
**MIP1B**	n.s.		n.s.		n.s.		n.s.		n.s.		n.s.	
**PDGFAA**	2.53	**0.01**	n.s.		n.s.		n.s.		-2.17	**0.03**	n.s.	
**PDGFBB**	2.19	**0.03**	n.s.		n.s.		n.s.		-2.00	**0.05**	n.s.	
**RANTES**	n.s.		n.s.		n.s.		n.s.		n.s.		n.s.	
**TGFA**	n.s.		n.s.		n.s.		n.s.		n.s.		n.s.	
**TNFA**	2.64	**0.009**	5.06	**< .0001**	4.14	**< .0001**	3.42	**0.0006**	2.37	**0.02**	n.s.	
**TNFB**	n.s.		-2.40	**0.02**	-2.27	**0.03**	n.s.		-2.03	**0.05**	n.s.	
**VEGF**	n.s.		n.s.		n.s.		n.s.		n.s.		n.s.	

*directionality of change goes with second group listed in each comparison (e.g., CD40L is elevated in the AUD relative to the Control group)

### Cytokine correlations

The functional significance of changes to peripheral cytokine levels was evaluated by exploring relationships with other blood markers; AUD-related variables (e.g., AUD onset age, lifetime alcohol consumption, days since last drink, scores on the AUD Identification Test [AUDIT], history of emergency room detoxifications/treatments, withdrawal scores); HIV-related variables (e.g., Karnofsky score, VACs Index, HIV onset age, HIV duration, CD4 cell count, CD4 cell count nadir, viral, AIDS-defining events); and general demographic variables such as body mass index (BMI) and smoking status.

**Fig 2:**
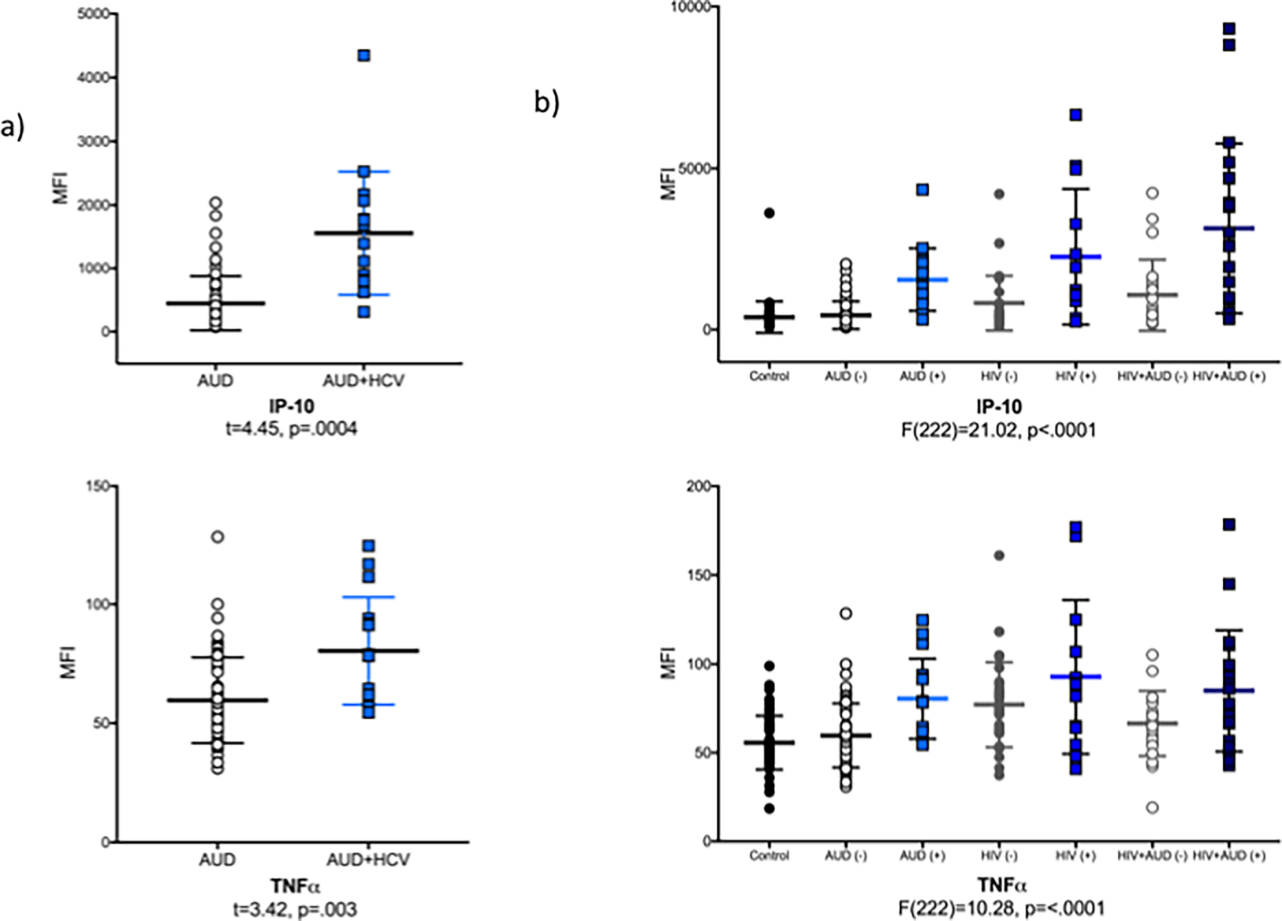
Scatter plots of Interferon γ-induced Protein 10 (IP-10) and Tumor Necrosis Factor α (TNFα) in **a)** the AUD group by HCV status (i.e., AUD without HCV: open black circles; AUD+HCV: blue squares) and **b)** all 4 study groups by HCV status (Control: black closed circles; AUD (-): AUD without HCV, black open circles; AUD (+): AUD +HCV, blue squares; HIV (-): HIV without HCV, gray closed circles; HIV (+): HIV+HCV: dark blue squares; HIV+AUD (-): HIV+AUD without HCV, gray open circles; HIV+AUD (+): HIV+AUD+HCV, midnight squares).

In the AUD group only, IP-10 (p = .0004) and TNFα (p = .003) levels were higher in AUD HCV-seropositive relative to AUD HCV-seronegative participants (**Fig 2A**). In addition, IP-10 levels correlated with depressive symptoms (i.e., total BDI-II score: ρ = .26, p = .03), alkaline phosphatase (AP: ρ = .28, p = .01), AST (ρ = .54, p < .0001), ALT (ρ = .42, p = .0002), and gamma-glutamyltransferase (GGT: ρ = .45, p < .0001). Similarly, TNFα levels in the AUD group only correlated with AP (ρ = .23, p = .04), AST (ρ = .31, p = .0006), ALT (ρ = .32, p = .005), and GGT (ρ = .35, p = .002). Of all the relationships evaluated between remaining cytokines affected by an AUD diagnosis and other blood markers, AUD-related variables, or general demographic variables, the only other significant correlation was between higher withdrawal scores and lower levels of MIP-1α (ρ = -.28, p = .01).

In the HIV group only, IP-10 (p = .03) levels were also higher in HIV+HCV co-infected relative to mono-infected HIV seropositive individuals; MCP-1 (ρ = .46, p = .003), IP-10 (ρ = .42, p = .008), and TNFα (ρ = .42, p = .008) levels positively correlated with the VACS index; and GCSF levels were lower with longer HIV duration (ρ = -.31, p = .04). No other relationships emerged in the HIV group between affected cytokines and relevant variables.

In the HIV+AUD group, IP-10 (p = .002) and TNFα (p = .04) levels were higher in the HIV+AUD group with HCV relative to the group without HCV. Furthermore, in the HIV+AUD group alone, lower Karnofsky scores were associated with lower levels of IFN- γ and higher levels of IP-10 and TNFα. No other relationships emerged in the HIV+AUD group between affected cytokines and relevant variables.

An AIC to predict IP-10 levels across the 3 patient groups including all associated variables (i.e., HCV status, BDI score, AP, AST, ALT, GGT, VACS index, and Karnofsky score) highlighted GGT levels, Karnofsky score, VACS index, and HCV status. A multiple regression including these 4 variables was significant (F(143) = 19.52, p < .0001), explained 36% of the variance in IP-10 levels, and was driven by the HCV status (p < .0001). Indeed, HCV status alone explained 26% of the variance in IP-10 levels. For TNFα, a similar AIC (excluding BDI scores) highlighted AST levels, VACS index, and HCV status. A multiple regression including these 3 variables was significant (F(148) = 18.10, p < .0001), explained 27% of the variance in TNFα levels, and was driven by the VACS index (p < .0001).

### Relevance of HCV infection

**Fig 3:**
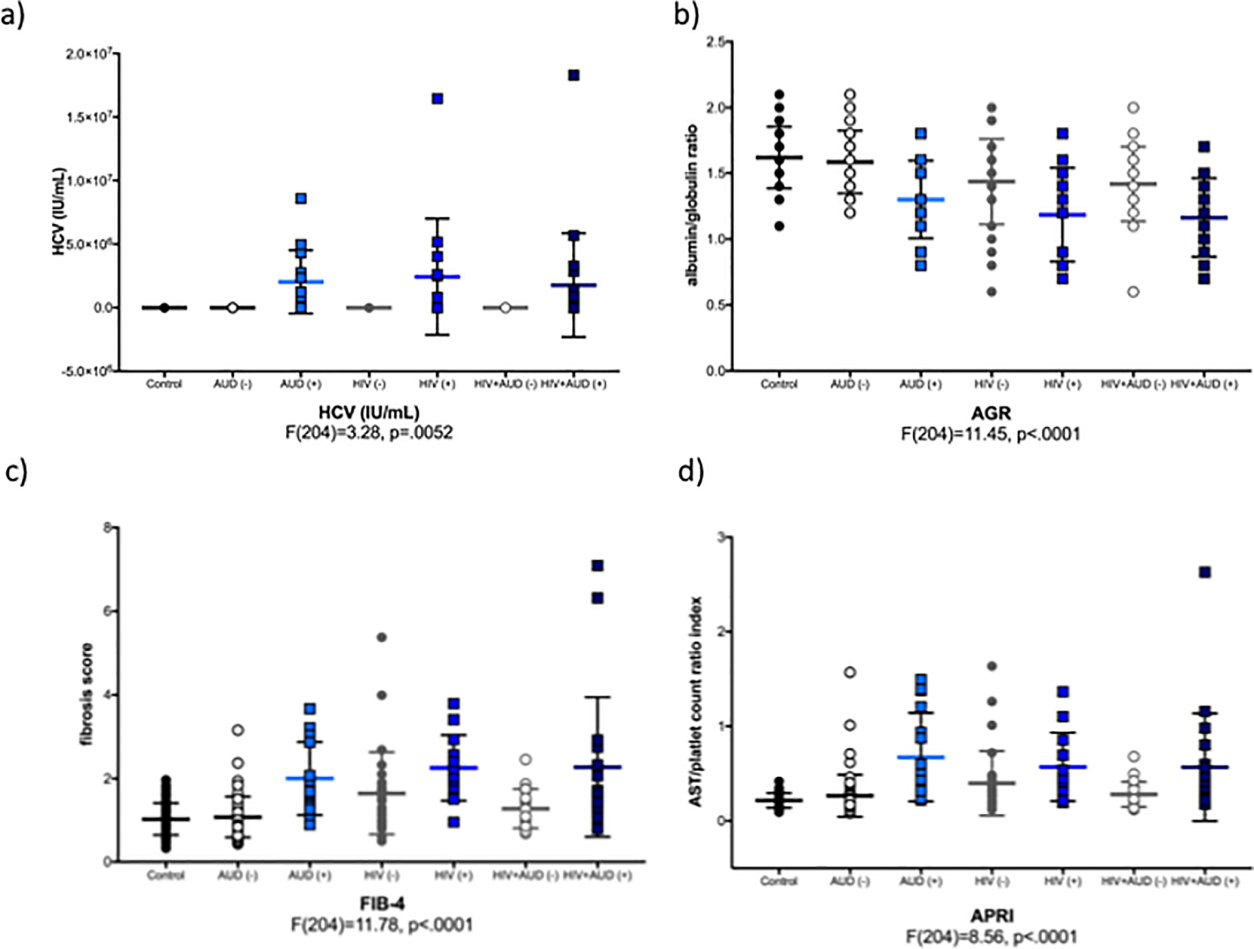
Scatter plots of **a)** HCV viral load, **b)** albumin/globulin ratio (AGR), **c)** fibrosis score (FIB-4), and **d)** AST/platelet count ratio index (APRI) in the 4 study groups by HCV status (see legend to Fig 2 for details).

**Fig 4:**
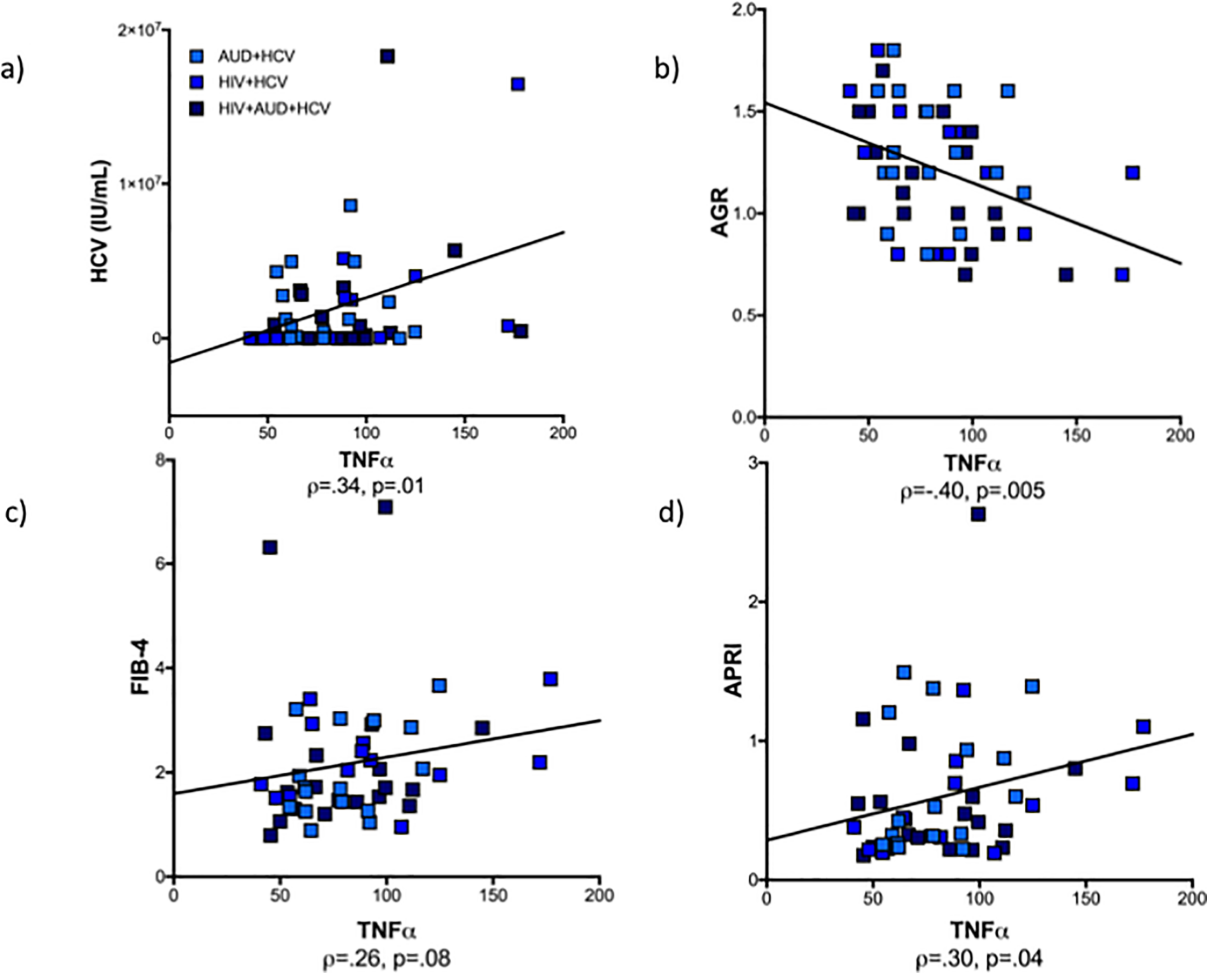
Correlations in the HCV-seropositive patient subgroups between TNFα levels and **a)** HCV viral load, **b)** albumin/globulin ratio (AGR), **c)** fibrosis score (FIB-4), and **d)** AST/platelet count ratio index (APRI).

To pursue the potential effect of HCV on group differences, the initial 4 groups were subdivided by HCV status into 7 groups (control, and each of the 3 patient groups (AUD, HIV, HIV+AUD) with and without HCV). The patient subgroups infected with HCV had elevated IP-10 (F(222) = 21.02, p < .0001) and TNFα (F(222) = 10.28, p = < .0001) levels (**Fig 2B**). Thus, HCV-related measures were also evaluated for their effects on IP-10 and TNFα. **Fig 3A** demonstrates the presence of HCV viral load (International Units/mL) in patient subgroups with HCV. Additional 7-group ANOVAs demonstrated that the albumin/globulin ratio (AGR: F(204) = 11.45, p < .0001; **Fig 3B**) was low and FIB-4 (F(204) = 11.78, p < .0001; **Fig 3C**) and APRI (F(204) = 8.56, p < .0001; **Fig 3D**) scores were high in the HCV-infected subgroups. These indices of liver compromise (HCV viral load ρ = .34, p = .01; AGR ρ = -.40, p = .005; FIB-4 ρ = .26, p = .08; APRI ρ = .30, p = .04) correlated with TNFα levels in the HCV-seropositive patient subgroups (**Fig 4**). Correlations were similar for IP-10 (HCV viral load ρ = .43, p = .002; AGR ρ = -.26, p = .08; FIB-4 ρ = .21, p = .16; APRI ρ = .26, p = .08). Levels of IP-10 and TNFα were not related to self-report of treatment for HCV.

## Discussion

The hypothesized role of the innate and adaptive immune systems in mood, psychiatric, and neurodegenerative disorders has gained significant support in the literature e.g., [143–145]. The aim of the current study was to determine whether uncomplicated alcoholism, that is, AUD in the absence of diagnosable medical concomitants, is associated with peripheral cytokine levels, in the context of similarly measured analytes in HIV, a disorder with a clearly demonstrated inflammatory component. Our results show that elevations in peripheral cytokines are associated not with an AUD diagnosis, but were associated with co-occurring HCV infection in abstinent drinkers.

A number of additional findings support the necessity of HCV infection to increase proinflammatory cytokine levels in AUD and HAART-controlled HIV subjects. When the HIV groups were similarly sub-categorized based on HCV status, the subgroups co-infected with HCV showed marked elevations in IP-10 and TNFα. Furthermore, across the HCV-infected individuals from the 3 patient groups, HCV viral load correlated with IP-10 and TNFα levels.

To provide further evidence that liver status affects cytokine levels in this population, we found that the albumin/globulin ratio (AGR) discriminated individuals with HCV relative to those without HCV. This comports with the literature demonstrating that low serum albumin levels can be used to predict HCV infection [146] and that albumin levels may be an important mortality risk factor for those co-infected with HIV and HCV [147]. In the HCV-infected patient subgroups included in this study, lower AGR correlated with higher IP-10 and TNFα levels.

We additionally calculated two descriptive, noninvasive indices of liver fibrosis [140]. FIB-4 scores (<1.45 absent; 1.45–3.25 intermediate fibrosis; >3.25 advanced fibrosis) have been used to predict and stage liver fibrosis in HCV and other forms of liver disease [128, 141, 142]. Our HCV patient subgroups had FIB-4 scores ranging from 2.00–2.27, indicating the presence of intermediate stage liver fibrosis. FIB-4 scores correlated weakly with IP-10 and TNFα levels in the subgroups with HCV infection.

In a meta-analysis of 40 studies, investigators concluded that an AST/platelet count ratio index (APRI) score greater than 1.0 had a sensitivity of 76% and specificity of 72% for predicting cirrhosis [148]: low APRI scores (<0.5) have negative predictive value to rule out cirrhosis; high APRI scores (> 1.5) have positive predictive value to diagnose cirrhosis. The APRI estimate has been used as alternative to frequent liver biopsies in HCV to detect and stage fibrosis e.g., [149–152]. The HCV subgroups included in this study had midrange APRI scores (0.56–0.67) and thus, cirrhosis cannot be ruled out. APRI scores also correlated with IP-10 and TNFα levels in subgroups with HCV infection.

The current finding of elevated TNFα in AUD + HCV is consistent with reports of hospitalized alcoholics showing correlations between high TNFα levels and liver dysfunction [89, 101, 107]. Alcoholic hepatitis is known to be associated with upregulation of serum cytokines [153, 154] and alcohol-related liver cirrhosis has been specifically associated with high TNFα levels [155], which have been used to predict mortality in alcoholic liver disease [156]. Our study contrasts with those reporting effects of “uncomplicated” AUD on increasing proinflammatory cytokine levels in notable ways: in the previously published studies, AUD subjects were currently actively drinking or hospitalized for drinking at the time of blood draw; and liver integrity, including presence of HCV, was not described e.g., [96–99, 108].

Our findings also comport with the HIV+HCV literature that has demonstrated a particular sensitivity of IP-10 levels to co-infection [157–159] and relationships between IP-10 levels and biomarkers of liver disease [160–162]. As has previously been suggested, however, alcoholism does not appear to have an effect on cytokine responses in HIV+HCV comorbidity [163].

A limitation of the current study was the absence of a non-AUD, HCV seropositive control group. It is our intention to include this comparison group in future studies. Further absent is a comparison group of recently detoxified alcoholics, who might be more likely to exhibit abnormal levels of cytokines cf., [96–99, 108].

In conclusion, this study reports elevations in TNFα in AUD individuals abstinent at examination that occurred only in the presence of HCV infection and suggests that changes in TNFα levels in AUD are dependent on derangement of liver function and not on alcohol-related variables. This finding encourages a careful characterization of alcoholics in human studies, including documentation of comorbid infections that can affect peripherally circulating levels of cytokines and chemokines.

## Supporting information

S1 Table Cytokine levels (pg/mL) in the 4 study groups: Median, 25%, and 75% quartiles. (XLSX)

S2 Table Corrected cytokine levels* in the 4 study groups: Mean±SD and ANOVA results. (XLSX)

S3 Table Two-group t-test comparisons of corrected cytokine levels. (XLSX)
